# Supplementary material for: Certified algorithms for equilibrium states of local quantum Hamiltonians
Source: Nat Commun. 2024 Aug 27;15:7394. doi: 10.1038/s41467-024-51592-3 (PMC11349919; doi:10.1038/s41467-024-51592-3)
Supplement: Supplementary file 1 — Supplementary Information [file 41467_2024_51592_MOESM1_ESM.pdf]

Certified algorithms for equilibrium states of local quantum Hamiltonians
Supplementary information

Hamza Fawzi Omar Fawzi Samuel O. Scalet

1 Preliminaries

We consider spin systems on a (potentially infinite) discrete set of sites Γ and we adopt the operator algebraic point of view. We describe the setup briefly here, and we refer to [1] or [2, Section 6.2] for more details.

For any site $x \in \Gamma$, the local Hilbert space $\mathcal{H}_x \simeq \mathbb{C}^d$ has dimension d. The Hilbert space associated to a finite subset $X \subset \Gamma$ is the tensor product $\mathcal{H}_X = \otimes_{x \in X} \mathcal{H}_x$, and we let $\mathcal{A}_X = \mathcal{B}(\mathcal{H}_X)$ be the algebra of observables supported on X. Let

$$\mathcal{A}_{\text{loc}} = \bigcup_{\substack{X \subset \Gamma \\ X \text{ finite}}} \mathcal{A}_X \tag{1}$$

be the set of local observables, and $\mathcal{A} = \overline{\mathcal{A}_{\text{loc}}}$ be its completion with respect to the operator norm, i.e., $\mathcal{A}$ is the C^*-algebra of quasi-local observables. (Obviously, when Γ is finite then $\mathcal{A}_{\text{loc}} = \mathcal{A} = \overline{\mathcal{A}}$ is the full algebra of complex matrices of size $d^{|\Gamma|} \times d^{|\Gamma|}$.) A state is a linear functional $\omega : \mathcal{A} \to \mathbb{C}$ such that $\omega(1) = 1$, $\omega(a^*) = \overline{\omega(a)}$, and $\omega(a^*a) \geq 0$ for all $a \in \mathcal{A}$.

Hamiltonians Consider a Hamiltonian H, which can be formally written as

$$H = \sum_{\substack{X \subset \Gamma \\ X \text{ finite}}} h_X \tag{2}$$

where $h_X \in \mathcal{A}_X$ are the local interaction terms. We assume that the Hamiltonian H is local, i.e., there exists a constant r such that h_X is nonzero only for X having size at most r, for every site $x \in \Gamma$, $|\{X \subset \Gamma : x \in X, h_X \neq 0\}| \leq r$, and that $\|h_X\| \leq 1$ for all X. In the case of the infinite lattice $\Gamma = \mathbb{Z}^D$, we say that the Hamiltonian is translation-invariant if $h_{X+x} = \tau_x(h_X)$ for all $X \subset \mathbb{Z}^D$ and $x \in \mathbb{Z}^D$, where $\tau_x : \mathcal{A}_X \to \mathcal{A}_{X+x}$ is the translation operator by x. If Λ is a finite subset of Γ, we let

$$H_\Lambda = \sum_{X \subset \Lambda} h_X \in \mathcal{A}_\Lambda \tag{3}$$

and

$$\tilde{H}_\Lambda = \sum_{X \cap \Lambda \neq \emptyset} h_X \in \mathcal{A}_{\overline{\Lambda}} \tag{4}$$

where $\overline{\Lambda} = \Lambda \cup \partial^{ex}\Lambda$, and $\partial^{ex}\Lambda$ is the external boundary of Λ, i.e.,

$$\partial^{ex}\Lambda = \{y \in \Lambda^c : \exists Y \subset \mathbb{Z}^D, h_Y \neq 0, y \in Y, \Lambda \cap Y \neq \emptyset\}. \tag{5}$$

When the lattice Γ is infinite, H is not a well-defined element of the algebra $\mathcal{A}$; however for any $a \in \mathcal{A}_{\text{loc}}$, we formally write $[H, a]$ for the well-defined element of $\mathcal{A}_{\text{loc}}$ that is equal to

$$[H, a] = [\tilde{H}_\Lambda, a], \tag{6}$$

when $a \in \mathcal{A}_\Lambda$.

Ground states A state $\omega : \mathcal{A} \to \mathbb{C}$ is called a *ground state* of H if

$$\omega(a^*[H, a]) \geq 0 \quad \forall a \in \mathcal{A}_{\text{loc}}. \tag{7}$$

In the case of finite systems, this condition is equivalent to saying that the density matrix of ω is supported on the eigenspace of H of minimal eigenvalue. To see this, we note that for finite systems, states are described by density matrices $\omega(a) = \text{tr}[\rho a]$. Choosing the operators a in the above definition for a given eigenbasis of the Hamiltonian e_i as $e_j e_i^*$, we see that

$$\rho_{ii} E_j - \rho_{ii} E_i \geq 0 \tag{8}$$

where we denote matrix elements ρ_{ij} in the energy eigenbasis and the corresponding eigenvalues by E_i. This is fulfilled only if $\rho_{ii} = 0$ for all i corresponding to eigenvectors that are not in the ground-space sector. By writing

$$\rho = \begin{pmatrix} A & B \\ B^* & C \end{pmatrix} \tag{9}$$

where the blocks correspond to the ground-space and its orthogonal complement, we can see that C has nonpositive diagonal entries, but is also positive semidefinite and thereby 0. By the Schur complement lemma we can then conclude that $B = 0$ and thereby ρ is supported on the ground space.

Remark 1.1 (Relation to thermodynamic limit)**.** Consider the case of a Hamiltonian on the infinite lattice $\Gamma = \mathbb{Z}^D$. It is immediate to verify that the limit of ground states of finite Hamiltonians $\tilde{H}_\Lambda$ of increasing size will automatically satisfy condition (7). Actually this is even true for any choice of "boundary condition" Δ_{Λ^c} acting outside Λ. Indeed, one can easily show that if ω is such that

$$\omega(a) = \lim_{\Lambda \uparrow \mathbb{Z}^D} \langle \Phi^\Lambda | a | \Phi^\Lambda \rangle \tag{10}$$

where for each Λ, Φ^Λ is a ground state of the finite Hamiltonian $\tilde{H}_\Lambda + \Delta_{\Lambda^c}$, then ω will satisfy (7). Imposing such arbitrary boundary conditions while taking the limit may actually be needed in order to capture the different ground states in the thermodynamic limit.

To illustrate this, consider the following one-dimensional classical system with three states $\{0, 1, 2\}$ per site given by the Hamiltonian

$$h_{i,i+1}(s, s') = \begin{cases} 0 & \text{if } s = s' \in \{0, 1\} \\ 2 & \text{if } (s, s') \in \{(0,1), (1,0), (2,2), (2,1), (0,2), (1,2)\} \\ -1 & \text{if } s = 2, s' = 0. \end{cases} \tag{11}$$

For the finite Hamiltonian $\tilde{H}_\Lambda$ with $\Lambda = \{-\ell, \dots, \ell\}$, the unique ground state is the sequence $(2, 0, \dots, 0)$ with energy -1. This makes it also the ground state of the infinite system corresponding to the limit with open boundary condition. Importantly, note that for the observable $O = |0\rangle\langle 0|$ at site 0, we have that for any $\ell \geq 1$, its value in the ground state of H_Λ is 1. However, another valid ground state of the infinite chain is the all-1 state. For this ground state in the thermodynamic limit, the observable takes a value of 0. It is realized by adding a boundary term $h_{\ell+1}(s) = -2\delta_{1,s}$ as it lowers the energy of the all-1 configuration below the open boundary ground state.

Beyond this simple but artificial example, there are many more classical and quantum models exhibiting an analogous effect that are of current interest in quantum many-body physics. Due to the more involved definitions and solutions of these models we refrain from giving them explicitly. Besides ubiquitous examples like the 1D Ising model which can be solved, in [3] the example of degenerate ground states of the quantum double model and an explicit construction of boundary conditions is given.

Thermal equilibrium states For a finite system, the Gibbs state at inverse temperature $\beta = 1/T \geq 0$ is given by the density matrix $\rho = e^{-\beta H}/\operatorname{tr} e^{-\beta H}$. An alternative characterization is via the so-called Kubo-Martin-Schwinger (KMS) conditions, which takes the form (for finite systems)

$$\omega(ba) = \omega(ae^{-\beta H}be^{\beta H}) \quad \forall a, b \in \mathcal{A}_{\text{loc}}. \tag{12}$$

It is easy to verify that the Gibbs state $\omega(a) = \operatorname{tr}(e^{-\beta H}a)/\operatorname{tr} e^{-\beta H}$ satisfies the equality conditions above, and that it is also the unique such state. To state the KMS-conditions for infinite systems, one first needs to introduce the time evolution operator

$$\alpha_t(a) = \lim_{\Lambda\uparrow\Gamma} e^{iH_\Lambda t} a e^{-iH_\Lambda t} \qquad \forall a \in \mathcal{A}_{\text{loc}}, t \in \mathbb{R} \tag{13}$$

where the limit is taken over a suitable sequence of finite subsets Λ. For the local Hamiltonian interactions we consider here, α_t exists and is strongly continuous [2, Theorem 6.2.4]. For a given time-evolution operator, there is a dense subset $\mathcal{A}_\alpha \subset \mathcal{A}$ such that $F(\alpha_t(a))$ is entire for all $a \in \mathcal{A}_\alpha$ and bounded linear functionals F [4, Proposition 2.5.22]. We say that a state $\omega : \mathcal{A} \to \mathbb{C}$ is a β-KMS state if [2, Definition 5.3.1]

$$\omega(ba) = \omega(a\alpha_{i\beta}(b)) \quad \forall a, b \in \mathcal{A}_\alpha. \tag{14}$$

In fact, it is sufficient to ensure this condition for any dense α-invariant *-subalgebra of $\mathcal{A}_\alpha$. The set of β-KMS states will be denoted by $\mathcal{G}_\beta$:

$$\mathcal{G}_\beta = \{\omega : \mathcal{A} \to \mathbb{C} : \omega(1) = 1,\ \omega(a^*a) \geq 0\ \forall a \in \mathcal{A}_{\text{loc}},\ \text{and (14)}\}. \tag{15}$$

For translation-invariant Hamiltonians on the lattice $\Gamma = \mathbb{Z}^D$ we will be mostly interested in translation-invariant KMS-states

$$\mathcal{G}_\beta^{\text{TI}} = \left\{\omega : \mathcal{A} \to \mathbb{C} : \omega \in \mathcal{G}_\beta \text{ and } \omega(a) = \omega(\tau_x(a))\ \forall a \in \mathcal{A}_{\text{loc}}, x \in \mathbb{Z}^D\right\}. \tag{16}$$

The set $\mathcal{G}_\beta$ (and $\mathcal{G}_\beta^{\text{TI}}$ for translation-invariant Hamiltonians) is nonempty for all $\beta \geq 0$. This can be shown by taking an appropriate limit of finite Gibbs states on a sequence of $\Lambda \uparrow \Gamma$. An important aspect of infinite systems (i.e., systems in the thermodynamic limit) is that the set of thermal equilibrium states $\mathcal{G}_\beta$ is not necessarily reduced to a singleton. The existence of many β-KMS states signals the presence of different thermodynamic phases.

2 Convex relaxations via energy-entropy balance inequalities

Our goal is to obtain a convex relaxation for the marginals of equilibrium states $\omega \in \mathcal{G}_\beta$. A natural way to obtain a convex relaxation is to impose only a subset of the β-KMS equality conditions (14) (which corresponds to (12) for finite systems). The problem with the KMS linear equality constraints is that even if a, b are local observables, the observable $a\alpha_{i\beta}(b)$ is in general not local (unless, say, the Hamiltonian is commuting).

2.1 Energy-entropy balance inequalities

The energy-entropy inequalities is a family of convex inequalities that characterize β-KMS states and that only involve local observables of ω. They were first derived by Araki and Sewell in [5].

Theorem 2.1 (Energy-entropy balance (EEB) inequalities, see [2, Theorem 5.3.15])**.** *Let H be a local Hamiltonian. Then $\omega : \mathcal{A} \to \mathbb{C}$ is a β-KMS state if, and only if, it satisfies*[1]

$$\omega([H, a]) = 0 \quad \forall a \in \mathcal{A}_{loc}, \tag{17}$$

$$\omega(a^*a) \log\left(\frac{\omega(a^*a)}{\omega(aa^*)}\right) \leq \beta\omega(a^*[H, a]) \quad \forall a \in \mathcal{A}_{loc}. \tag{18}$$

[1] The function $x\log(x/y)$ is defined as equal to 0 if $x = 0$ and $+\infty$ if $x > 0$ and $y = 0$.

Remark 2.2. The cited theorem requires the conditions to hold for all elements in the domain of the generator δ of the time-evolution operator α_t. On $\mathcal{A}_{\rm loc}$ this operator takes values $\delta(a) = i[H, a]$. It is, however, defined on a larger domain given by the closure of this operator, i.e., the commutator formula on $\mathcal{A}_{\rm loc}$ is a *core* for δ. By the definition of the closure of an operator, $a \in \mathcal{A}$ is said to be in the domain of δ, i.e., $a \in D(\delta)$ if there exists a sequence $a_i \in \mathcal{A}_{\rm loc}$ such that $a_i \to a$ *and* $\delta(a_i) \to \delta(a)$. Due to the latter, it is sufficient to impose the EEB inequalities on $\mathcal{A}_{\rm loc}$ only, despite the lack of continuity of δ.

The convexity of the constraints (18) in ω follow from convexity of the function $(x, y) \mapsto x \log(x/y)$. Furthermore if H is a local Hamiltonian, and a is a local operator, then the constraints (2.1) only involve local observables of ω. Note that for $\beta = +\infty$ (zero temperature), the constraint (18) reduces to the inequality (7) which characterizes the ground states of H.

Remark 2.3 (Relation to thermodynamic limit)**.** Using conditions (2.1) one can show that any limit of finite Gibbs states $\exp(-\beta H_\Lambda)/ \operatorname{tr} \exp(-\beta H_\Lambda)$ as $\Lambda \uparrow \Gamma$ will be a valid β-KMS state. Actually the same is true for any sequence of finite Gibbs states of $H_\Lambda + \Delta_{\Lambda^c}$ where Δ_{Λ^c} is an arbitrary term acting only on the boundary of Λ.

Similarly to the example for ground-states in Remark 1.1, we demonstrate how different thermal states can arise from different choices of boundary conditions. Consider the 2D Ising model at some temperature $0 < T < T_c$ (where T_c is the critical temperature), and let for any $\ell \in \mathbb{N}$, $\rho_{\ell,\beta}$ be the finite Gibbs state on a region $\{-\ell, \dots, \ell\}^2$ with open boundary condition. Then by the spin-flip symmetry of the Ising Hamiltonian, we know that $\operatorname{tr}(Z_x \rho_{\ell,\beta}) = 0$ for any site x in the region $\{-\ell, \dots, \ell\}^2$. Thus this means that for any limit ω_β of the finite Gibbs state with open boundary condition, we will have $\omega_\beta(Z_x) = 0$. It is known however that for the 2D Ising model below the critical temperature, there are two (extremal) infinite-volume Gibbs states ω_β^+ and ω_β^- such that $\omega_\beta^-(Z_x) < 0 < \omega_\beta^+(Z_x)$, which can be reached by taking limits of finite Hamiltonians with fixed boundary condition, i.e., aligned spins on the boundary [6].

2.2 Convex relaxations

The EEB inequalities above can be used to obtain rigorous lower and upper bounds on the value of any observable on thermal states via convex optimization. Let $(\Lambda_\ell)_{\ell \in \mathbb{N}}$ be an increasing sequence of subsets of Γ such that the support of O is contained in Λ_0, and such that $\Lambda_\ell \uparrow \Gamma$ as $\ell \to \infty$. To simplify the presentation, we will further assume that $\overline{\Lambda_{\ell-1}} \subset \Lambda_\ell$ for all ℓ, where, as before, $\overline{\Lambda} = \Lambda \cup \partial^{ex} \Lambda$ (see (5)). For example, if $\Gamma = \mathbb{Z}^D$ and H is a nearest-neighbor Hamiltonian and O is supported on site 0, one can take $\Lambda_\ell = \{-\ell, \dots, \ell\}^D$. For any ℓ, let $\mathcal{A}_\ell \subset \mathcal{A}$ be the subalgebra of observables supported on Λ_ℓ. Consider the optimization problem

$$\min_{\tilde{\omega}:\mathcal{A}_\ell \to \mathbb{C}}/\max \quad \tilde{\omega}(O) \tag{19}$$

$$\text{s.t.} \quad \tilde{\omega}(1) = 1 \tag{20}$$

$$\tilde{\omega}(a^* a) \geq 0 \quad \forall a \in \mathcal{A}_\ell \tag{21}$$

$$\tilde{\omega}([H, a]) = 0 \ \forall a \in \mathcal{A}_{\ell-1} \tag{22}$$

$$\tilde{\omega}(a^* a) \log \left(\frac{\tilde{\omega}(a^* a)}{\tilde{\omega}(a a^*)} \right) \leq \beta \tilde{\omega}(a^* [H, a]) \quad \forall a \in \mathcal{A}_{\ell-1}. \tag{23}$$

The feasible set of this convex optimization problem is an *outer relaxation* for the set of Λ_ℓ-marginals of thermal equilibrium states in $\mathcal{G}_\beta$, i.e., the optimization variable takes values in the set of states on $\mathcal{A}_\ell$. This relaxation is obtained by imposing the EEB inequalities on the finite-dimensional subspace $\mathcal{A}_{\ell-1}$, in addition to the normalization and positivity constraints (20), (21). Besides the linear equations (20) and (22), the constraint (21) can be encoded as the positivity of a Hermitian matrix of size $|\Lambda_\ell| \times |\Lambda_\ell|$. However, the last set of constraints (23) form an infinite set of scalar inequalities, and it is unclear how to formulate them in a compact way. This is due to the lack of linearity in the inequality. Imposing the

inequality for a basis of $\mathcal{A}_{\ell-1}$is not sufficient to conclude it on all elements of $\mathcal{A}_{\ell-1}$, which is an infinite set. Clearly one can sample certain observables $a_i \in \mathcal{A}_{\ell-1}$ $(i = 1, \ldots, m)$ and impose the m scalar inequalities, however, this poses the question of how to choose these a_i's.

Matrix EEB inequality We introduce the following matrix version of the EEB inequality which allows us to impose the EEB inequality for all observables a in a finite-dimensional subspace. The matrix EEB inequality makes use of the matrix relative entropy function [7, 8] which is defined as

$$D_{op}(A\|B) = A^{1/2}\log(A^{1/2}B^{-1}A^{1/2})A^{1/2} \tag{24}$$

for any two positive semidefinite matrices $A, B \succeq 0$ such that $A \ll B$ (i.e., $\operatorname{im} A \subset \operatorname{im} B$). The function D_{op} is the operator perspective [9, 10] of the negative logarithm function (which is operator convex) and satisfies the following properties, see [8]:

- Homogeneity: $D_{op}(\lambda A\|\lambda B) = \lambda D_{op}(A\|B)$ for $\lambda > 0$
- Operator convexity[2]: $D_{op}(\sum_k A_k\| \sum_k B_k) \preceq \sum_k D_{op}(A_k\|B_k)$
- Transformer inequality: For any rectangular matrix K,

$$D_{op}(K^*AK\|K^*BK) \preceq K^*D_{op}(A\|B)K. \tag{25}$$

Furthermore, the function D_{op} is closed, in the sense that $\{(A, B, T) : A \ll B \text{ and } D_{op}(A\|B) \preceq T\}$ is a closed convex set [11, Theorem B.1]. Convex optimization problems involving the matrix relative entropy can be solved using interior-point methods [11] or via semidefinite programming approximations [12].

Theorem 2.4 (Matrix EEB inequality)**.** *Let H be a local Hamiltonian and $\omega : \mathcal{A} \to \mathbb{C}$ be a β-KMS state. Let $a_1, \ldots, a_m \in \mathcal{A}_{loc}$ and define the $m \times m$ matrices*

$$\begin{aligned} A_{ij} &= \omega(a_i^* a_j) \\ B_{ij} &= \omega(a_j a_i^*) \\ C_{ij} &= \omega(a_i^*[H, a_j]). \end{aligned} \tag{26}$$

Then A, B, C are Hermitian, A, B are positive semidefinite with $A \ll B$ and $D_{op}(A\|B) \preceq \beta C$.

Proof. It is clear that A, B are Hermitian and that they are positive semidefinite. That $A \ll B$ follows from the fact that for a KMS state $\omega(bb^*) = 0 \implies \omega(b^*b) = 0$ for any $b \in \mathcal{A}$. (This can be seen e.g., from (18)). To see that C is Hermitian, note that since ω is a KMS state we have $\omega([H, b]) = 0$ for any $b \in \mathcal{A}_{\text{loc}}$ (see (17)). Then

$$C_{ij} = \omega(a_i^*[H, a_j]) = \omega(a_i^*(Ha_j - a_jH)) = \omega(a_i^*Ha_j - Ha_i^*a_j) = \omega([a_i^*, H]a_j) = \overline{\omega(a_j^*[H, a_i])} = \overline{C_{ji}}. \tag{27}$$

We now prove the inequality $D_{op}(A\|B) \preceq \beta C$. We present the proof here in the finite-dimensional case. We treat the general case in Appendix A. We thus assume that $\mathcal{A}$ is a finite-dimensional matrix algebra. Let $L : \mathcal{A} \to \mathcal{A}$ be the commutator operator with the Hamiltonian H, i.e., $L(a) = [H, a]$. The map L is self-adjoint with respect to the Hilbert-Schmidt inner product. Observe that for any $a \in \mathcal{A}$, $e^{-\beta L}(a) = e^{-\beta H}ae^{\beta H}$ (this can be easily seen e.g., by checking that $a(\beta) := e^{-\beta H}ae^{\beta H}$ is the solution of the ODE $a'(\beta) = -L(a(\beta))$ with $a(0) = a$.) Let $L = \sum_k \lambda_k P_k$ be a spectral decomposition of L. By the KMS condition we have

$$B_{ij} = \omega(a_j a_i^*) = \omega(a_i^* e^{-\beta L}(a_j)) = \sum_k e^{-\beta\lambda_k}\omega(a_i^* P_k(a_j)) = \sum_k e^{-\beta\lambda_k} A_{ij}^{(k)} \tag{28}$$

[2] For homogeneous functions, convexity is equivalent to subadditivity

where we let $A^{(k)}_{ij} = \omega(a_i^* P_k(a_j))$. Since $\sum_k P_k = I$ and $\sum_k \lambda_k P_k = L$, we have

$$\sum_k A^{(k)} = A \quad \text{and} \quad \sum_k \lambda_k A^{(k)} = C. \tag{29}$$

Then we have

$$D_{op}(A\|B) = D_{op}\left(\sum_k A^{(k)} \| \sum_k e^{-\beta\lambda_k} A^{(k)}\right) \preceq \sum_k D_{op}(A^{(k)} \| e^{-\beta\lambda_k} A^{(k)}) = \sum_k \beta\lambda_k A^{(k)} = \beta C \tag{30}$$

as desired. □

A convex relaxation using the matrix relative entropy We now consider the modified convex optimization problem

$$\min_{\tilde\omega:\mathcal{A}_\ell\to\mathbb{C}}/\max \quad \tilde\omega(O) \tag{31}$$

$$\text{s.t.} \quad \tilde\omega(1) = 1 \tag{32}$$

$$\tilde\omega(a^*a) \geq 0 \quad \forall a \in \mathcal{A}_\ell \tag{33}$$

$$\tilde\omega([H,a]) = 0 \; \forall a \in \mathcal{A}_{\ell-1} \tag{34}$$

$$D_{op}\left([\tilde\omega(a_i^* a_j)]_{ij} \, , \, [\tilde\omega(a_j a_i^*)]_{ij} \right) \preceq \beta[\tilde\omega(a_i^*[H,a_j])]_{ij}. \tag{35}$$

where in the last constraint, $\{a_i\}$ is a basis of $\mathcal{A}_{\ell-1}$. One can show, using (25), that the infinite number of scalar constraints (23) are implied by the finite positive semidefinite constraint (35). We prove this in the next proposition.

Proposition 2.5. *Consider the optimization problem (2.2) and assume $\{a_i\}$ ($i = 1,\dots,m$) is a basis of $\mathcal{A}_{\ell-1}$. Then the constraints (23) are all implied by the matrix inequality (35).*

Proof. Let a be an arbitrary element of $\mathcal{A}_{\ell-1}$, and let $a = \sum_i x_i a_i$, with $x_i \in \mathbb{C}$, be its decomposition in the basis $\{a_i\}$. Also let A, B, C be the matrices defined in (26), so that the inequality (35) can be written as $D_{op}(A\|B) \preceq \beta C$. If we let $x = (x_1,\dots,x_m) \in \mathbb{C}^m$, then note that

$$\begin{aligned} x^*Ax &= \sum_{ij} \overline{x_i} x_j \omega(a_i^* a_j) = \omega(a^*a) \\ x^*Bx &= \sum_{ij} \overline{x_i} x_j \omega(a_j a_i^*) = \omega(aa^*) \\ x^*Cx &= \sum_{ij} \overline{x_i} x_j \omega(a_i^*[H,a_j]) = \omega(a^*[H,a]). \end{aligned} \tag{36}$$

It follows from (25) that

$$\omega(a^*a)\log\left(\frac{\omega(a^*a)}{\omega(aa^*)}\right) = D_{op}(x^*Ax\|x^*Bx) \leq x^*D_{op}(A\|B)x \leq \beta x^*Cx = \beta\omega(a^*[H,a]) \tag{37}$$

as desired. □

2.3 Convergence

Let $(\mathsf{P}_\ell^{\min})$ and $(\mathsf{P}_\ell^{\max})$ be respectively the minimization and maximization problems (2.2), and let $\mathsf{p}_\ell^{\min} \leq \mathsf{p}_\ell^{\max}$ be their optimal values. The next theorem shows the asymptotic convergence of $\mathsf{p}_\ell^{\min}$ and $\mathsf{p}_\ell^{\max}$ to $\langle O\rangle_\beta^{\min}$ and $\langle O\rangle_\beta^{\max}$ respectively.

Theorem 2.6. *Let H be a local Hamiltonian, $O \in \mathcal{A}_{loc}$ a local observable, and $\beta \in [0, +\infty]$. Define*

$$[\langle O\rangle_\beta^{\min}, \langle O\rangle_\beta^{\max}] = \{\omega(O) : \omega \in \mathcal{G}_\beta\}. \tag{38}$$

Then $\mathsf{p}_\ell^{\min} \uparrow \langle O\rangle_\beta^{\min}$ and $\mathsf{p}_\ell^{\max} \downarrow \langle O\rangle_\beta^{\max}$ as $\ell \to \infty$.

Proof. Note that $\mathcal{G}_\beta$ is convex and weak-* compact [2, Theorem 5.3.30] and so $\{\omega(O) : \omega \in \mathcal{G}_\beta\}$ is a closed interval.

If $\omega \in \mathcal{G}_\beta$, then for any ℓ, its restriction to $\mathcal{A}_\ell$ is feasible for the optimization problems (2.2). Thus this means that $\mathsf{p}_\ell^{\min} \leq \langle O\rangle_\beta^{\min} \leq \langle O\rangle_\beta^{\max} \leq \mathsf{p}_\ell^{\max}$ for any ℓ. Furthermore, observe that $\mathsf{p}_\ell^{\min}$ is monotonic nondecreasing, and $\mathsf{p}_\ell^{\max}$ is monotone nonincreasing. Indeed assume $\tilde{\omega}_\ell : \mathcal{A}_\ell \to \mathbb{C}$ is feasible for (2.2) and consider its restriction to $\mathcal{A}_k$ for $k < \ell$. We want to show that it is feasible for the level k of the relaxation (2.2). It is immediate that the restriction satisfies the constraints (32)-(34) at the level k of the relaxation. To prove (35) we use the transformer inequality (25). Let $\{b_s\}$ be a basis of $\mathcal{A}_{k-1}$ and note that we can express each b_s as a linear combination of the $a_i \in \mathcal{A}_{\ell-1}$, i.e., $b_s = \sum_i K_{is} a_i$ for some $K_{is} \in \mathbb{C}$. As such we have

$$\tilde{\omega}(b_s^* b_t) = \sum_{ij} \overline{K_{is}} K_{jt} \tilde{\omega}(a_i^* a_j) \tag{39}$$

so that we can write $[\tilde{\omega}(b_s^* b_t)]_{s,t} = K^*[\tilde{\omega}(a_i^* a_j)]_{i,j} K$ and similarly for $[\tilde{\omega}(b_t b_s^*)]_{s,t}$. Hence we get

$$\begin{aligned} D_{op}([\tilde{\omega}(b_s^* b_t)]_{s,t}\ ,\ [\tilde{\omega}(b_t b_s^*)]_{s,t}) &= D_{op}(K^*[\tilde{\omega}(a_i^* a_j)]_{i,j} K\ , K^*[\tilde{\omega}(a_j a_i^*)]_{i,j} K) \\ &\preceq K^* D_{op}([\tilde{\omega}(a_i^* a_j)]_{i,j}\ ,\ [\tilde{\omega}(a_j a_i^*)]_{i,j}) K \\ &\preceq \beta K^*[\tilde{\omega}(a_i^*[H, a_j])]_{i,j} K \\ &= \beta[\tilde{\omega}(b_s^*[H, b_t])]_{s,t}, \end{aligned} \tag{40}$$

as desired.

Since $(\mathsf{p}_\ell^{\min})$ and $(\mathsf{p}_\ell^{\max})$ are monotonic and bounded they must have limits $\mathsf{p}_\infty^{\min} := \lim \mathsf{p}_\ell^{\min}$ and $\mathsf{p}_\infty^{\max} := \lim \mathsf{p}_\ell^{\max}$ respectively. We want to show that $\mathsf{p}_\infty^{\min} = \langle O\rangle_\beta^{\min}$ and $\mathsf{p}_\infty^{\max} = \langle O\rangle_\beta^{\max}$.

For each ℓ, let $\tilde{\omega}_\ell : \mathcal{A}_\ell \to \mathbb{C}$ be a feasible solution to (2.2) such that $\mathsf{p}_\ell^{\min} \leq \tilde{\omega}_\ell(O) \leq \mathsf{p}_\ell^{\min} + 1/\ell$ (if the minimum value of the optimization problem is attained, we simply take $\tilde{\omega}_\ell$ the optimal solution which satisfies $\tilde{\omega}_\ell(O) = \mathsf{p}_\ell^{\min}$). We can extend $\tilde{\omega}_\ell$ to a state on the full C^*-algebra $\mathcal{A}$ [4, Proposition 2.3.24]. We now have a sequence of states $\tilde{\omega}_\ell$ on $\mathcal{A}$. Since the set of states on $\mathcal{A}$ is weak-* compact [4, Theorem 2.3.15] we can extract from $\tilde{\omega}_\ell$ a subsequence $\tilde{\omega}_{n(\ell)}$ that weak-* converges to a state $\tilde{\omega}$ on $\mathcal{A}$.

We now show that $\tilde{\omega}$ is a β-KMS state by showing that the conditions (2.1) hold. Let $a \in \mathcal{A}_{\text{loc}}$. Since a is a local observable we have $a \in \mathcal{A}_{n(\ell)-1}$ for all large enough ℓ. Hence this implies that

$$\begin{aligned} &\tilde{\omega}_{n(\ell)}([H, a]) = 0 \\ &\tilde{\omega}_{n(\ell)}(a^* a) \log\left(\frac{\tilde{\omega}_{n(\ell)}(a^* a)}{\tilde{\omega}_{n(\ell)}(a a^*)}\right) \leq \beta \tilde{\omega}_{n(\ell)}(a^*[H, a]) \end{aligned} \tag{41}$$

for all large enough ℓ. By taking the limit $\ell \to \infty$ this shows that $\tilde{\omega}$ satisfies the conditions (2.1) and is thus a β-KMS state. Finally note that

$$\tilde{\omega}(O) = \lim_\ell \tilde{\omega}_{n(\ell)}(O) \leq \lim_\ell \mathsf{p}_{n(\ell)}^{\min} + 1/n(\ell) = \mathsf{p}_\infty^{\min}. \tag{42}$$

Since $\mathsf{p}_\infty^{\min} \leq \langle O\rangle_\beta^{\min} \leq \tilde{\omega}(O)$ we necessarily get equality $\mathsf{p}_\infty^{\min} = \langle O\rangle_\beta^{\min}$. The same argument can be used to show that $\mathsf{p}_\infty^{\max} = \langle O\rangle_\beta^{\max}$. □

Translation-invariant Hamiltonians We now prove the main theorem, which we restate here.

Theorem 2.7 (Certified algorithms for expectation values of equilibrium states)**.** *Let $\Lambda_0 \subset \Lambda_1 \subset \cdots \subset \Gamma$ be an increasing sequence such that the support of the local observable O is contained in Λ_0. For any $\ell \in \mathbb{N}$, let $\mathsf{p}_\ell^{\min}$ and $\mathsf{p}_\ell^{\max}$ be respectively the minimum and maximum values of the convex optimization problems (2.2) with $\Lambda = \Lambda_\ell$. Then we have*

$$\mathsf{p}_\ell^{\min} \leq \langle O \rangle_\beta^{\min} \leq \langle O \rangle_\beta^{\max} \leq \mathsf{p}_\ell^{\max}. \tag{43}$$

Furthermore, $\mathsf{p}_\ell^{\min} \uparrow \langle O \rangle_\beta^{\min}$ and $\mathsf{p}_\ell^{\max} \downarrow \langle O \rangle_\beta^{\max}$ as $\ell \to \infty$.

For translation-invariant Hamiltonians, one can adapt the hierarchy (2.2) so that the resulting sequences $(\mathsf{p}_\ell^{\min,\mathrm{TI}})$ and $(\mathsf{p}_\ell^{\max,\mathrm{TI}})$ converge to $\langle O \rangle^{\min,\mathrm{TI}}$ and $\langle O \rangle^{\max,\mathrm{TI}}$ respectively. It suffices to add the following translation-invariance linear constraint in the convex optimization problem:

$$\tilde{\omega}(\tau_x(a)) = \tilde{\omega}(a) \quad \forall a \in \mathcal{A}_\ell \ \forall x \in \mathbb{Z}^D \text{ s.t. } \tau_x(a) \in \mathcal{A}_\ell. \tag{44}$$

To prove convergence, one simply needs to show (using the same notations as in the proof of Theorem 2.6) that $\tilde{\omega} = \text{weak*-}\lim \tilde{\omega}_{n(\ell)}$ is translation-invariant. To do this, let $a \in \mathcal{A}_{\mathrm{loc}}$ and $x \in \mathbb{Z}^D$. Since a is a local operator, we know that $a, \tau_x(a) \in \mathcal{A}_{n(\ell)}$ for all large enough ℓ. This means that

$$\tilde{\omega}(\tau_x(a)) = \lim_\ell \tilde{\omega}_{n(\ell)}(\tau_x(a)) = \lim_\ell \tilde{\omega}_{n(\ell)}(a) = \tilde{\omega}(a) \tag{45}$$

where the middle equality follows from the translation-invariant constraint (44) imposed in the optimization problem. This is true for all $a \in \mathcal{A}_{\mathrm{loc}}$ and $x \in \mathbb{Z}^D$, and so $\tilde{\omega}$ is translation-invariant.

2.4 Decidability

It has been shown in [13] that computing the phase diagram of a 2D nearest neighbour translation-invariant Hamiltonian in the case $\beta = \infty$ is undecidable. This is shown for both a definition of phase in terms of gapped and gapless Hamiltonians as well as in terms of an order parameter. It is interesting to compare the latter with our result. More precisely, the construction of the authors gives a Hamiltonian (described by a nearest neighbour interaction $h(n)$) for every input n to a universal Turing machine and a local observable O such that

- If the Turing machine halts on input n then for any sequence of ground states $|\psi_L\rangle$ of the Hamiltonian on an $L \times L$ square with open boundary conditions $\lim_{L\to\infty} \langle \psi_L | O | \psi_L \rangle = 0$.
- If the Turing machine does not halt on input n, then for any sequence of ground states $|\psi_L\rangle$ of the Hamiltonian on an $L \times L$ square with open boundary conditions $\lim_{L\to\infty} \langle \psi_L | O | \psi_L \rangle = 1$.

It is interesting to compare this to our algorithm by asking what the output of our algorithm would be given the construction above as its input. We leave a full resolution of this question to future work. However, in light of the proven undecidability we can rule out that there exists a constant $\varepsilon < 1/2$ such that the interval provided by our algorithm is below ε for all Hamiltonians arising from halting instances and above $1 - \varepsilon$ for all non-halting ones.

We can however say the following to partially explain how the proof in [13] would not extend to arbitrary boundary conditions. The limit values in this construction crucially depend on the choice of boundary conditions. While the boundary condition is not mentioned explicitly as it is always kept open, in fact a technique from [14] is used to encode a boundary term corresponding to an energy shift of a Hamiltonian into the translation-invariant interaction term. Before this shift the construction yields a nonnegative ground-state energy in the nonhalting case and a negative one in the halting case. Adding this energy shift and adding a subspace $\{|0\rangle\}$ with a trivial product state with zero energy to the Hamiltonian,

the ground state in the nonhalting case will be given by this product state. The observable $O = |0\rangle\langle 0|$ is then given by a projector onto this subspace.

The definition of ground-states in our work is different and in fact the set of equilibrium states $\mathcal{G}_\infty$ includes the limits of ground-states of Hamiltonians for *any* boundary condition, see Remark 1.1. In this version, as opposed to the one above, our hierarchies do prove the decidability of the equilibrium observable problem as given in the following definition. This means that the problem solution is defined over the set of all boundary conditions, not that the problem can be decided for every choice of boundary condition.

Definition 2.8.

Equilibrium Observable Problem	
**Input:**	Local dimension d, local Hamiltonian term h with rational coefficients, local observable O with rational coefficients, rational number a, temperature $\beta \in [0, +\infty]$
**Promise:**	Either $\langle O\rangle_\beta^{\min,\mathrm{TI}} > a$ or $\langle O\rangle_\beta^{\max,\mathrm{TI}} < a$
**Question:**	Output YES if $\langle O\rangle_\beta^{\min,\mathrm{TI}} > a$, NO otherwise

Supplementary Box 1: Definition of the Equilibrium Observable Problem

Theorem 2.9. *The Equilibrium Observable Problem is decidable.*

Proof. Let us start with the case $\beta = \infty$. The algorithm consists of iterating over $\ell = 2, 3, \ldots$ and alternating between the minimization and maximization of the convex relaxations (2.2) with the translation-invariant constraint (44). Note that for the ground-state Eq. (35) reduces to $0 \preceq \beta[\tilde{\omega}(a_i^*[H, a_j])]_{ij}$, such that the problem becomes an SDP in rational coefficients that can be solved exactly. More specifically, we use quantifier elimination [15, 16] to decide whether the hierarchy augmented by $\tilde{\omega}(O) > a$ or augmented by $\tilde{\omega}(O) < a$ is feasible. The algorithm stops as soon as one of them becomes infeasible and outputs NO or YES respectively. Due to Theorem 2.7 this is guaranteed to happen at sufficiently large ℓ.

In the case $\beta < \infty$, we face additional difficulties due to the matrix logarithm, as our convex relaxation is no longer an SDP. We will use SDP approximations of the logarithm, however, those have convergence guarantees that are only uniform on intervals that are bounded away from 0. A way to deal with this issue is to strengthen our convex relaxation by an *a priori* lower bound on the moment matrices derived from an analysis of the Hamiltonian. This is proven in the following Lemma by adapting a result from [17] to the infinite system moment problem setting.

Lemma 2.10. *For a given local translation-invariant Hamiltonian H on $\mathbb{Z}^D$ and temperature β, there exists a KMS-state $\omega \in \mathcal{G}_\beta$ such that the following is true: for any finite set $\Lambda \subset \mathbb{Z}^D$, there exists an explicit constant $C_\Lambda > 0$ depending on β, the locality and norm of the Hamiltonian, and Λ, such that the marginal $\rho_\Lambda \in \mathcal{A}_\Lambda$ of ω, defined by*

$$\mathrm{tr}[\rho_\Lambda a] = \omega(a) \qquad \forall a \in \mathcal{A}_\Lambda \tag{46}$$

is lower bounded

$$\rho_\Lambda \succeq C_\Lambda. \tag{47}$$

Proof. Without loss of generality we assume that the Hamiltonian is defined on qubits since general qudit systems can be embedded by encoding each qudit in a Hilbert space $\mathbb{C}^{2^{m^D}}$ such that $d \leq 2^{m^D}$, corresponding to a hypercube of spins. This results in a Hamiltonian with higher but still finite range.

In [17, Corollary 2.14] it is shown that for a given qubit Hamiltonian H_V on a finite set V and for a fixed $\Lambda \subset V$ there exist explicit constants c_Λ, c'_Λ such that the following is true: for any Hermitian observable $X = \sum_i x_i a_i$ supported on $\Lambda \subset V$ (here, $x_i \in \mathbb{R}$ and a_i is the orthonormal Pauli basis supported on Λ) we have

$$\operatorname{tr}[X^2 \tilde{\rho}_V] \geq \exp(-c_\Lambda - \beta c'_\Lambda) \max x_i^2, \tag{48}$$

where $\tilde{\rho}_V = e^{-\beta H_V} / \operatorname{tr} e^{-\beta H_V}$ is the Gibbs state at inverse temperature β for the given finite Hamiltonian H_V. Crucially, the constants c_Λ, c'_Λ do not depend on $|V|$, but only on the locality of the Hamiltonian and the degree of its so-called dual interaction graph, which are fixed.[3] If we take the weak*-limit of Gibbs states $\tilde{\rho}_V$ on increasing system sizes $V \uparrow \mathbb{Z}^D$ (taking an appropriate subsequence to ensure convergence, see [2, Proposition 6.2.15]) we get a KMS-state ω which satisfies

$$\omega\left(X^2\right) \geq \exp(-c_\Lambda - \beta c'_\Lambda) \max_i x_i^2 \tag{49}$$

for all $X \in \mathcal{A}_\Lambda$, where the x_i are the coefficients of X in the Pauli basis of $\mathcal{A}_\Lambda$.

Now let ρ_Λ be the marginal of ω on Λ, and let X be the projector onto a one-dimensional eigenspace of ρ_Λ with lowest eigenvalue. Note that the x_i are real as X is a Hermitian operator and that

$$\max_i x_i^2 \geq \frac{1}{4^{|\Lambda|}} \|(x_i)_i\|_2^2 = \frac{1}{4^{|\Lambda|}} \|X\|_F^2 = \frac{1}{4^{|\Lambda|}}. \tag{50}$$

Thereby, we conclude

$$\rho_\Lambda \succeq \operatorname{tr}\left[\rho_\Lambda X^2\right] = \omega\left(X^2\right) \geq \frac{1}{4^{|\Lambda|}} \exp(-c_\Lambda - \beta c'_\Lambda) =: C_\Lambda. \tag{51}$$

□

We now choose the basis of $\mathcal{A}_\Lambda$ in our convex relaxation (2.2) to be orthonormal. We see that the above Lemma proves a lower bound on the lowest eigenvalue of the moment matrix ω that corresponds to the KMS-state given in the Lemma:

$$\min_{\|x\|_2^2=1} \langle x|[\tilde{\omega}(a_i^* a_j)]_{ij}|x\rangle = \min_{\|x\|_2^2=1} \sum_{ij} \overline{x_i} x_j \omega(a_i^* a_j) = \operatorname{tr}[\rho X^* X] \geq C_{\Lambda_\ell} \operatorname{tr}[X^* X] = C_{\Lambda_\ell} \tag{52}$$

and similarly for $[\tilde{\omega}(a_j a_i^*)]_{ij}$. We use this to strengthen our convex relaxation (2.2) by adding the contraints

$$\begin{aligned} [\tilde{\omega}(a_i^* a_j)]_{ij} &\succeq C'_{\Lambda_\ell} > 0, \\ [\tilde{\omega}(a_j a_i^*)]_{ij} &\succeq C'_{\Lambda_\ell} > 0 \end{aligned} \tag{53}$$

where C'_{Λ_ℓ} is a rational lower bound to C_{Λ_ℓ}. Note that Lemma 2.10 need not hold for KMS states that arise from different boundary conditions as those might come with different constants c, c'. In the promise problem setting this is not relevant though as the relaxation still contains at least the open boundary Gibbs state and thereby the respective SDP problem is always feasible. Therefore, the optimal values of the modified problems are two sequences $\mathsf{p}_\ell^{\min} \leq \mathsf{p}_\ell'^{\min} \leq \mathsf{p}_\ell'^{\max} \leq \mathsf{p}_\ell^{\max}$. Note that monotonicity and thereby convergence are no longer guaranteed for $\mathsf{p}_\ell'^{\min}, \mathsf{p}_\ell'^{\max}$. However, we only need that the optimal values exceed the decision threshold, which is guaranteed by convergence of $\mathsf{p}_\ell^{\min}, \mathsf{p}_\ell^{\max}$ and the above inequality.

After this modification, since the moment matrix is bounded away from zero and upper bounded by 1, the eigenvalues in the argument of the logarithm for the matrix relative entropy are contained in an interval $[a, 1/a]$ for some $a > 0$. We now introduce a semidefinite approximation of D_{op} from [12]. For any integer $m \geq 1$ let $h_m(x) = 2^m \left(x^{1/2^m} - 1\right)$ which satisfy $\log(x) \leq h_{m+1}(x) \leq h_m(x)$ and note that

[3] The dual interaction graph has nodes for each Hamiltonian term and edges between terms that overlap

$h_m \to \log$ uniformly on any compact interval in $(0, \infty)$ since $0 \le h_m(x) - \log(x) \le 2^{-m}[(x-1)^2 + (x^{-1}-1)^2]$ for all $x > 0$. Consider the *noncommutative perspective* [9, 10] of $-h_m$ which we denote by $D_{op}^{[m]}$:

$$D_{op}^{[m]}(X, Y) := P_{-h_m}(X, Y) = -X^{1/2} h_m(X^{-1/2} Y X^{-1/2}) X^{1/2}. \tag{54}$$

Then $D_{op}^{[m]}$ is jointly convex in (X, Y), has a finite semidefinite programming formulation [12], and satisfies $D_{op}^{[m]}(X\|Y) \preceq D_{op}(X\|Y)$ since $-h_m \le -\log$. By changing the function D_{op} in the entropy constraint (35) by $D_{op}^{[m]}$ we get a semidefinite program $(\mathsf{P}'^{\min}_{\ell,m})$ with rational coefficients whose optimal value satisfies $\mathsf{p}'^{\min}_{\ell,m} \le \mathsf{p}'^{\min}_{\ell}$ and analogously for the upper bounds.

Furthermore using the constraint (53), the uniform convergence of h_m to log, and the continuity of D_{op} on positive definite matrices, we know that $\mathsf{p}'^{\min}_{\ell,m} \uparrow \mathsf{p}'^{\min}_{\ell}$ and similarly $\mathsf{p}'^{\max}_{\ell,m} \downarrow \mathsf{p}'^{\max}_{\ell}$ as $m \uparrow \infty$. By choosing an exhaustive sequence (ℓ_i, m_i) of $\mathbb{N}^+ \times \mathbb{N}^+$ and deciding the two feasibility problems as in the ground-state case we obtain an algorithm that decides the problem in finite time.

□

2.5 Commuting Hamiltonians and the high-temperature regime

In this section we focus on commuting Hamiltonians H which satisfy $[h_X, h_Y] = 0$ for any $X, Y \subset \Gamma$. For concreteness, we choose to focus on H translation-invariant on $\Gamma = \mathbb{Z}^D$. For such Hamiltonians one can define a convex relaxation for the set of thermal equilibrium states directly from the KMS equality condition. Indeed, in this case if $b \in \mathcal{A}_\Lambda \subset \mathcal{A}_{\text{loc}}$ where Λ is finite, then

$$\alpha_t(b) = \lim_{\Lambda' \uparrow \mathbb{Z}^D} e^{itH_{\Lambda'}} b e^{-itH_{\Lambda'}} = e^{it\tilde{H}_\Lambda} b e^{-it\tilde{H}_\Lambda} \tag{55}$$

where $\tilde{H}_\Lambda$ is as defined in (4). Clearly, in this case $\alpha_t(b)$ can be extended to an entire function for all $b \in \mathcal{A}_{\text{loc}}$. As such, the β-KMS condition for a state ω can be written as

$$\omega(ba) = \omega(a e^{-\beta \tilde{H}_\Lambda} b e^{\beta \tilde{H}_\Lambda}) \qquad \forall a \in \mathcal{A}_{\bar{\Lambda}},\ b \in \mathcal{A}_\Lambda \tag{56}$$

for all $\Lambda \subset \mathbb{Z}^D$ finite. (Recall that $\overline{\Lambda} = \Lambda \cup \partial^{ex}\Lambda$ where $\partial^{ex}\Lambda$ is the external boundary of Λ.) A consequence of these KMS conditions[4] is that

$$\omega([a, h_X]) = 0 \ \ \forall a \in \mathcal{A}_{\bar{\Lambda}},\ \forall X \subset \bar{\Lambda} \tag{57}$$

for all $\Lambda \subset \mathbb{Z}^D$ finite. Given a fixed $\Lambda \subset \mathbb{Z}^D$ finite, let[5]

$$\begin{aligned} \mathcal{G}^{\text{comm}}_{\Lambda,\beta} = \Big\{ \omega : \mathcal{A}_{\bar{\Lambda}} \to \mathbb{C} \text{ s.t. } & \omega(1) = 1, \omega(a^* a) \ge 0 \ \ \forall a \in \mathcal{A}_{\bar{\Lambda}}, \\ & \omega(a) = \omega(\tau_x(a))\ \forall a \in \mathcal{A}_{\bar{\Lambda}}, x \in \mathbb{Z}^D \text{ s.t. } \tau_x(a) \in \mathcal{A}_{\bar{\Lambda}} \\ & \text{and (56) and (57)} \Big\}. \end{aligned} \tag{58}$$

Since (56) is bilinear in (a, b) it only needs to be imposed on a basis $\{a_i\}$ of $\mathcal{A}_{\bar{\Lambda}}$ and $\{b_j\}$ of $\mathcal{A}_\Lambda$. As such, the convex set (58) is the feasible set of a semidefinite program. It is an outer relaxation for the set of $\bar{\Lambda}$-marginals of β-KMS states.

Given an observable $O \in \mathcal{A}_\Lambda$, one can consider the convex optimization problems

$$\min / \max \ \{\tilde{\omega}(O) \text{ s.t. } \tilde{\omega} \in \mathcal{G}^{\text{comm}}_{\Lambda,\beta}\}. \tag{59}$$

[4] This is because $\alpha_t(h_X) = h_X$, since the Hamiltonian is commuting.

[5] Note that (56) for a fixed Λ only implies (57) for $X \subset \Lambda$ (and not $\bar{\Lambda}$). That's why we impose (57) explicitly.

The solution of this minimization (resp. maximization) problem is a *lower bound* on $\langle O\rangle_\beta^{\min,\mathrm{TI}}$ (resp. *upper bound* on $\langle O\rangle_\beta^{\max,\mathrm{TI}}$).

The asymptotic convergence of the hierarchy (59) for a sequence of increasing finite lattices Λ_ℓ to $\langle O\rangle_\beta^{\min,\mathrm{TI}}$ and $\langle O\rangle_\beta^{\max,\mathrm{TI}}$ can be proved in exactly the same way as in Theorem 2.6. For this hierarchy however we are also able to give a quantitative rate of convergence in the high temperature regime.

Theorem 2.11. *Assume that the Hamiltonian is translation-invariant, commuting and that the lattice dimension $D \le 2$. For $D = 1$ and $\beta_1 = \infty$ or for $D = 2$ and some $\beta_1 > 0$, we have for all $0 \le \beta < \beta_1$, and for any local observable O the following:*

- $\langle O\rangle_\beta^{\min,\mathrm{TI}} = \langle O\rangle_\beta^{\max,\mathrm{TI}} = \langle O\rangle_\beta^{\mathrm{TI}}$
- *If we let $\mathsf{p}_\ell^{\min}$ and $\mathsf{p}_\ell^{\max}$ be the optimal values of the convex relaxations (59) with $\Lambda = \Lambda_\ell = \{-\ell,\dots,\ell\}^D$, then $\mathsf{p}_\ell^{\min} \le \langle O\rangle_\beta^{\mathrm{TI}} \le \mathsf{p}_\ell^{\max}$, and furthermore*

$$\max\left\{\langle O\rangle_\beta^{\mathrm{TI}} - \mathsf{p}_\ell^{\min}, \mathsf{p}_\ell^{\max} - \langle O\rangle_\beta^{\mathrm{TI}}\right\} \le c_1\|O\|e^{-c_2\ell}. \tag{60}$$

for some constants $c_1, c_2 > 0$ depending on the interaction, temperature and the support of O.

Proof. We start with an important lemma showing that states in $\mathcal{G}_{\Lambda,\beta}^{\mathrm{comm}}$ can be expressed as a product of the local (i.e., finite) Gibbs state on Λ and a boundary term on $\partial^{ex}\Lambda$, after a suitable perturbation that eliminates the surface interaction terms. The statement is closely related to [2, Prop. 6.2.17]. The difference is that the state ω in the lemma below is not assumed to be a "full" KMS state, but just a state in $\mathcal{G}_{\Lambda,\beta}^{\mathrm{comm}}$.

Lemma 2.12. *Assume H is a commuting Hamiltonian. Let $\Lambda \subset \mathbb{Z}^D$ be a finite set, and let $\omega \in \mathcal{G}_{\Lambda,\beta}^{comm}$. Let*

$$W = \sum_{\substack{X\cap\Lambda\neq\emptyset\\ X\cap\Lambda^c\neq\emptyset}} h_X \in \mathcal{A}_{\bar\Lambda} \tag{61}$$

be the surface interaction term. Let

$$\varphi^\Lambda(x) = \frac{\mathrm{tr}(e^{-\beta H_\Lambda}x)}{\mathrm{tr}\, e^{-\beta H_\Lambda}}. \tag{62}$$

There exists a state $\hat\omega : \mathcal{A}_{\partial^{ex}\Lambda} \to \mathbb{C}$ on the boundary such that using the product state $\omega^W : \mathcal{A}_{\bar\Lambda} \to \mathbb{C}$ defined by

$$\omega^W(xc) = \varphi^\Lambda(x)\hat\omega(c) \tag{63}$$

we can express ω as a perturbation of the local Gibbs state with a boundary condition

$$\omega(O) = \frac{\omega^W(Oe^{-\beta W})}{\omega^W(e^{-\beta W})}. \tag{64}$$

Proof. We define

$$\omega^W(a) = \frac{\omega(e^{\beta W}a)}{\omega(e^{\beta W})} = \frac{\omega(ae^{\beta W})}{\omega(e^{\beta W})}, \tag{65}$$

where the equality follows using the β-KMS condition and the fact that H is commuting. We first prove that it has the claimed form, i.e., is product with the factor on Λ being φ^Λ. Let $a, b \in \mathcal{A}_\Lambda$ and $c \in \mathcal{A}_{\partial^{ex}\Lambda}$.

Then condition (56) tells us that

$$\begin{aligned} \omega^W(bac) &= \frac{1}{\omega(e^{\beta W})}\omega(bace^{\beta W}) \\ &= \frac{1}{\omega(e^{\beta W})}\omega(ace^{\beta W}e^{-\beta\tilde{H}_\Lambda}be^{\beta\tilde{H}_\Lambda}) \\ &= \frac{1}{\omega(e^{\beta W})}\omega(ace^{-\beta H_\Lambda}be^{\beta H_\Lambda}e^{\beta W}) \\ &= \omega^W(ace^{-\beta H_\Lambda}be^{\beta H_\Lambda}) \\ &= \omega^W(ae^{-\beta H_\Lambda}be^{\beta H_\Lambda}c) \end{aligned} \tag{66}$$

where in the last line we used the fact that $c \in \mathcal{A}_{\partial^{ex}\Lambda}$ and so it commutes with $e^{-\beta H_\Lambda}be^{\beta H_\Lambda} \in \mathcal{A}_\Lambda$.

From (66) we get that for any fixed $c \in \mathcal{A}_{\partial^{ex}\Lambda}$, the state $x \in \mathcal{A}_\Lambda \mapsto \omega^W(xc)/\omega^W(c)$ satisfies the KMS condition for the finite-dimensional Hamiltonian H_Λ. Since this is a finite system, this means that necessarily for any $x \in \mathcal{A}_\Lambda$,

$$\frac{\omega^W(xc)}{\omega^W(c)} = \frac{\mathrm{tr}(e^{-H_\Lambda}x)}{\mathrm{tr}\, e^{-H_\Lambda}}. \tag{67}$$

This proves that ω^W is product and has the marginal on Λ as claimed. We conclude by computing by

$$\omega(O) = \frac{\omega(O)}{\omega(e^{\beta W})}\frac{\omega(e^{\beta W})}{\omega(I)} = \frac{\omega^W(Oe^{-\beta W})}{\omega^W(e^{-\beta W})}, \tag{68}$$

using the definition of ω^W twice. This proves Lemma 2.12. □

We are now ready to prove exponential convergence of the convex optimization hierarchy to $\langle O\rangle_\beta$ for $0 \le \beta < \beta_1$. We start with the 1D case. Our main tool will be the decay of correlations and local indistinguishability of Gibbs states for one-dimensional chains [18]. Without loss of generality let us assume a 2-local interaction; the general case follows from blocking the sites first. Let $\omega \in \mathcal{G}^{\mathrm{comm}}_{\Lambda_\ell,\beta}$ and consider the perturbation ω^W from Lemma 2.12. The surface interaction term W has support on the sites $-(\ell+1), -\ell, \ell, \ell+1$ and $\|\exp(\pm\beta W)\| \le \exp(2\beta\|h\|)$ is bounded by a constant. We first apply the decay of correlations to bound the effect of the perturbation. From Lemma 2.12 we have

$$\omega(O) = \frac{\omega^W(Oe^{-\beta W})}{\omega^W(e^{-\beta W})}. \tag{69}$$

Let us introduce the operator $Y = (id_\Lambda \otimes \hat{\omega}_{\partial^{ex}\Lambda})(e^{-\beta W}) \in \mathcal{A}_\Lambda$, which satisfies $\|Y\| \le \exp(2\beta\|h\|)$. We further decompose $Y = Y_L Y_R$ where Y_L, Y_R are supported on $-\ell$ and ℓ respectively. We can write

$$\omega(O) = \frac{\varphi^\Lambda(OY)}{\varphi^\Lambda(Y)} \tag{70}$$

and thereby

$$\begin{aligned} |\omega(O) - \varphi^\Lambda(O)| \le{}& \frac{|\varphi^\Lambda(OY) - \varphi^\Lambda(OY_L)\varphi^\Lambda(Y_R)|}{\varphi^\Lambda(Y)} && (71)\\ &+ \frac{|\varphi^\Lambda(OY_L)\varphi^\Lambda(Y_R) - \varphi^\Lambda(Y_L)\varphi^\Lambda(Y_R)\varphi^\Lambda(O)|}{\varphi^\Lambda(Y)} && (72)\\ &+ \frac{|\varphi^\Lambda(O)\varphi^\Lambda(Y_L)\varphi^\Lambda(Y_R) - \varphi^\Lambda(O)\varphi^\Lambda(Y)|}{\varphi^\Lambda(Y)} && (73)\\ &\le \|O\|K'\exp(-\alpha\ell) && (74) \end{aligned}$$

where to each term we can apply the uniform clustering from [18, Theorem 6.2], which extends the decay of correlations already known from Araki's seminal paper [19] to the case of Gibbs states on finite intervals. The constant K' here comes from the interaction dependent constant from the cited result combined with the (constant) norms of Y^{-1}, Y_L, Y_R and the locality of O reducing the distance between the supports of O and Y. The constant α depends on the interaction.

We conclude by [20, Theorem 4.16 (ii)] (see also [19]), which proves that expectations in the global Gibbs state are approximated by local ones, with an error decaying exponentially with the distance to the boundary, i.e.,

$$|\varphi^{\Lambda}(O) - \langle O\rangle_{\beta}^{\mathrm{TI}}| \leq \|O\| K'' \exp(-\delta\ell). \tag{75}$$

Again, δ and K'' depend on the interaction and the latter in addition on the locality of O. This proves Theorem 2.11 in the case $D = 1$, for all finite $\beta \geq 0$.

In higher dimension we expect the theorem to break down for arbitrary temperatures due to the existence of phase transitions. Nevertheless, at sufficiently high temperatures, the two main ingredients, decay of correlations and local indistinguishability still hold. A technical problem we are facing is however the (super)exponential growth of $e^{\beta W}$ since the support and norm of W scale with ℓ^{D-1}, where ℓ is the distance between O and the boundary. In 2D, however, we can still trade off this exponential growth against the decay rates by choosing sufficiently high temperatures. We closely follow the proof of the 1D case. Again we define $Y = (id_{\Lambda} \otimes \hat{\omega}_{\partial^{ex}\Lambda})(e^{-\beta W}) \in \mathcal{A}_{\Lambda}$. This time, however, we can only bound $\|Y\|, \|Y^{-1}\| \leq \exp(\beta\ell r\|h\|)$, where r is a constant depending on the locality of the Hamiltonian. For the decay of correlations we can resort to the high temperature result in [21, Theorem 2]. Below a critical inverse temperature this gives

$$\begin{aligned} |\omega(O) - \varphi^{\Lambda}(O)| &= \frac{|\varphi^{\Lambda}(OY) - \varphi^{\Lambda}(O)\varphi^{\Lambda}(Y)|}{\varphi^{\Lambda}(Y)} && (76)\\ &\leq \|O\|\|Y\|\|Y^{-1}\| C \exp(-\gamma\ell) && (77)\\ &\leq \|O\| C \exp((2\beta r\|h\| - \gamma)\ell) && (78) \end{aligned}$$

where C and γ depend on the interaction and temperature. Note that γ grows unboundedly for $\beta \to 0$. By choosing β sufficiently small such that $2\beta r\|h\| < \gamma$ this implies exponential decay.

Finally, local indistinguishability is proven in [21, Corollary 2] again above the same critical temperature and with the same decay rate. The formulation proves convergence of marginals in 1-norm distance but by choosing the region to be the support of O the result for observables follows:

$$|\varphi^{\Lambda}(O) - \langle O\rangle_{\beta}^{\mathrm{TI}}| \leq \|O\| K'' \exp(-\gamma\ell) \tag{79}$$

□

Remark 2.13. We remark that in the proof above, we do not make explicit use of the uniqueness of the β-KMS state for $0 \leq \beta < \beta_1$. This uniqueness actually follows from the proof since it holds for any choice of KMS-state and any observable. The used results on decay of correlations based on cluster expansions are in fact the same techniques used to prove the uniqueness of the KMS-state at sufficiently high temperature.

3 Numerical experiments

We consider the one-dimensional transverse field Ising model

$$H = -\sum_{i\in\mathbb{Z}} Z_i Z_{i+1} + gX_i \tag{80}$$

where X_i and Z_i are the usual Pauli matrices at site i and $g \geq 0$. The model is exactly solvable [22], and the ground state exhibits a phase transition at $g = 1$. For $g < 1$, the ground space is degenerate, and for $g > 1$, the ground state is unique. The model is gapped for $g \neq 1$, and gapless at $g = 1$. There is no phase transition with respect to the temperature as this is a one-dimensional model.

Let $\mathcal{G}_{g,\beta}$ be the set of translation-invariant equilibrium states for the parameter g of the Hamiltonian, and at inverse temperature β. For $g < 1$, $|\mathcal{G}_{g,\infty}| > 1$, while for $g > 1$ or $\beta < \infty$ we have $|\mathcal{G}_{g,\beta}| = 1$.

We focus on two observables, namely the Z-magnetization per site, i.e., $O = Z_0$, and the nearest-neighbour correlation function $O = Z_0 Z_1$.

Magnetization When $g = 0$, the model is a simple one-dimensional Ising model, and there are two translation-invariant ground states, namely the all-up and all-down states. This means that at $g = 0$ the magnetization can take any value between -1 and $+1$. For general $g \geq 0$, it has been shown that [22, Eq. (3.12a)]

$$\{\omega(Z_0) : \omega \in \mathcal{G}_{g,\infty}\} = [-M_z(g), M_z(g)] \tag{81}$$

where

$$M_z(g) = \begin{cases} (1-g^2)^{1/8} & \text{if } g < 1 \\ 0 & \text{if } g \geq 1. \end{cases} \tag{82}$$

Table 1 shows the true value of the magnetization for $g = 0.8$ and $g = 1.2$, along with the bounds obtained from the convex relaxation (2.2). The relaxations were solved respectively with a 4-site and 5-site region Λ. (Note that the semidefinite program for a L-site region involves a positivity constraint of a matrix of size 4^{L-1}.) We see as expected that the bounds with $L = 5$ are tighter than the bounds for $L = 4$.

	$\beta = \infty$		$\beta = 1$	
	$g = 0.8$	$g = 1.2$	$g = 0.8$	$g = 1.2$
True	0.8801	0	0	0
$L = 5$ u.b.	0.8809	0.0444	0.0844	0.0692
$L = 4$ u.b.	0.8823	0.2813	0.2146	0.1814

Supplementary Table 1: Bounds on magnetization $\langle Z_0 \rangle_{g,\beta}$ of the 1D TF Ising Model for different parameters g and β. By symmetry of the model we have $\langle Z_0 \rangle_\beta^{\min} = -\langle Z_0 \rangle_\beta^{\max}$. In the table we only show $\langle Z_0 \rangle_\beta^{\max}$ and upper bounds obtained from the convex optimization approach.

Spin-spin correlation function Next, we consider the nearest-neighbour correlation function $O = Z_0 Z_1$. The exact value for all choices of g and $\beta \in [0, +\infty]$ is given by [23, Eq. (10.58)]:[6]

$$\langle Z_0 Z_1 \rangle_{g,\beta} = \frac{1}{\pi} \int_0^\pi \frac{1 + g\cos k}{\sqrt{1 + 2g\cos k + g^2}} \tanh(J\beta\sqrt{1 + 2g\cos k + g^2})dk. \tag{83}$$

Table 2 compares the bounds obtained from the convex relaxations with the true value. For this observable we see a bigger gap with the true value. We also notice that at $\beta = \infty$ (ground state), the upper bounds are much closer to the true value than the lower bounds.

For $\beta < \infty$ the optimization problems involving the matrix relative entropy were solved using the semidefinite programming approximations [12]. We used the Mosek interior-point solver [24] for all the computations, except for the case $L = 5, \beta < \infty$ where we used the first-order solver SCS [25].

[6] Here the interval reduces to a single point, even when the set of equilibrium states is degenerate for $g < 1$ and $\beta = \infty$.

	$\beta = \infty$		$\beta = 1$	
	$g = 0.8$	$g = 1.2$	$g = 0.8$	$g = 1.2$
$L = 4$ l.b.	0.2177	0.0716	0.3109	0.2843
$L = 5$ l.b.	0.4660	0.3697	0.4303	0.3735
True	0.8125	0.4685	0.6224	0.4941
$L = 5$ u.b.	0.8168	0.5239	0.7504	0.5937
$L = 4$ u.b.	0.8904	0.8201	0.7946	0.6458

Supplementary Table 2: Bounds on correlation $\langle Z_0 Z_1 \rangle_{g,\beta}$ of the 1D TF Ising Model for different parameters g and β.

A Proof of Theorem 2.4

Our proof is analogous to [2, Theorem 5.3.15]. Let $\{a_1, \dots, a_m\}$ be a finite subset of $\mathcal{A}_{\text{loc}}$. Let $(\mathcal{H}, \pi, \Omega)$ be the cyclic representation for ω of $\mathcal{A}$ [2, Corollary 2.3.16] and

$$U(t) = \int_{-\infty}^{\infty} e^{-ipt} dE(p) \tag{84}$$

the canonical unitary group implementing the time evolution α_t [2, Corollary 2.3.17] in its spectral decomposition. Analogously to [2, page 88], we can define the following positive $m \times m$ Hermitian matrix-valued measures μ and ν.

$$[\mu(\hat{f})]_{kl} = (2\pi)^{-1/2} \int_{-\infty}^{\infty} dt f(t) \omega(a_k^* \alpha_t(a_l)) = \int_{-\infty}^{\infty} \langle \pi(a_k)\Omega, dE(q)\pi(a_l)\Omega \rangle \hat{f}(q) dq \tag{85}$$

$$[\nu(\hat{f})]_{kl} = (2\pi)^{-1/2} \int_{-\infty}^{\infty} dt f(t) \omega(\alpha_t(a_l) a_k^*) = \int_{-\infty}^{\infty} \langle \pi(a_k)\Omega, dE(-q)\pi(a_l)\Omega \rangle \hat{f}(q) dq \tag{86}$$

Here, the first part of each definition assumes a compactly supported and infinitely differentiable function $\hat{f}$ and its inverse Fourier transform

$$f(z) = (2\pi)^{-1/2} \int_{\infty}^{\infty} \hat{f}(p) e^{ipz} dp, \tag{87}$$

whereas the second part of the definition of the measures defines their continuation to arbitrary bounded continuous functions $\hat{f}$.

We see from the second part that $[\mu(1)]_{kl} = \omega(a_k^* a_l) = A_{kl}$ and $\nu(1) = B$. Furthermore, we show that $d\nu(p) = e^{p\beta} d\mu(p)$ following the analogous statement in [2, Proposition 5.3.14]. We do this by computing for a compactly supported infinitely differentiable function $\hat{f}$

$$[\mu(\hat{f})]_{kl} = \int_{\infty}^{\infty} dt f(t) \omega(a_k^* \alpha_t(a_l)) \tag{88}$$

$$= \int_{\infty}^{\infty} dt f(t + i\beta) \omega(\alpha_t(a_l) a_k^*) = [\nu(k_\beta \hat{f})]_{kl} \tag{89}$$

where $k_\beta(p) = e^{-\beta p}$, also using the characterization of KMS-states from [2, Proposition 5.3.12].

We now prove the inequality $D_{op}(A\|B) \preceq \beta C$. Let γ be the positive real-valued measure defined by $d\gamma(p) = \text{tr}[d\mu(p)]$. Since the trace is faithful, the measure μ has a matrix-valued density with respect to γ, i.e., we can write $d\mu(p) = \tilde{A}(p) d\gamma(p)$ where $\tilde{A} : \mathbb{R} \to \mathbf{H}_+^m$ (see e.g., [26, Section 4]). Then we can write

$$\begin{aligned} D_{op}(A\|B) &= D_{op}\left(\int 1 d\mu(p) \| \int 1 d\nu(p) \right) \\ &= D_{op}\left(\int \tilde{A}(p) d\gamma(p) \| \int e^{p\beta} \tilde{A}(p) d\gamma(p) \right) \\ &\preceq \int D_{op}(\tilde{A}(p) \| e^{p\beta} \tilde{A}(p)) d\gamma(p) \\ &= \beta \int -p\tilde{A}(p) d\gamma(p) = \beta \int -p d\mu(p) = C. \end{aligned} \tag{90}$$

References

[1] Nachtergaele, B. Quantum spin systems. In *Encyclopedia of Mathematical Physics*, 295–301 (Academic Press, Oxford, 2006). URL https://www.sciencedirect.com/science/article/pii/B0125126662000055.

[2] Bratteli, O. & Robinson, D. W. *Operator Algebras and Quantum Statistical Mechanics 2* (Springer, 1997), 2nd edn.

[3] Cha, M., Naaijkens, P. & Nachtergaele, B. The complete set of infinite volume ground states for Kitaev's Abelian quantum double models. *Communications in Mathematical Physics* **357**, 125–157 (2017). URL http://dx.doi.org/10.1007/s00220-017-2989-4.

[4] Bratteli, O. & Robinson, D. W. *Operator Algebras and Quantum Statistical Mechanics 1* (Springer, 1987), 2nd edn.

[5] Araki, H. & Sewell, G. L. KMS conditions and local thermodynamical stability of quantum lattice systems. *Communications in Mathematical Physics* **52**, 103–109 (1977).

[6] Friedli, S. & Velenik, Y. *Statistical mechanics of lattice systems: a concrete mathematical introduction* (Cambridge University Press, 2017).

[7] Fujii, J. I. & Kamei, E. Relative operator entropy in noncommutative information theory. *Math. Japon* **34**, 341–348 (1989).

[8] Fujii, J. I., Fujii, M. & Seo, Y. An extension of the Kubo-Ando theory: solidarities. *Math. Japon* **35**, 387–396 (1990).

[9] Effros, E. G. A matrix convexity approach to some celebrated quantum inequalities. *Proc. Natl. Acad. Sci. USA* **106**, 1006–1008 (2009).

[10] Ebadian, A., Nikoufar, I. & Gordji, M. E. Perspectives of matrix convex functions. *Proc. Natl. Acad. Sci. USA* **108**, 7313–7314 (2011).

[11] Fawzi, H. & Saunderson, J. Optimal self-concordant barriers for quantum relative entropies. *SIAM Journal on Optimization* **33**, 2858–2884 (2023).

[12] Fawzi, H., Saunderson, J. & Parrilo, P. A. Semidefinite approximations of the matrix logarithm. *Foundations of Computational Mathematics* **19**, 259–296 (2019).

[13] Bausch, J., Cubitt, T. S. & Watson, J. D. Uncomputability of phase diagrams. *Nature Communications* **12**, 452 (2021).

[14] Gottesman, D. & Irani, S. The quantum and classical complexity of translationally invariant tiling and Hamiltonian problems. In *2009 50th Annual IEEE Symposium on Foundations of Computer Science*, 95–104 (IEEE, 2009).

[15] Anai, H. On solving semidefinite programming by quantifier elimination. In *Proceedings of the 1998 American Control Conference. ACC (IEEE Cat. No.98CH36207)*, vol. 5, 2814–2818 vol.5 (1998).

[16] Basu, S., Pollack, R. & Roy, M.-F. *Algorithms in Real Algebraic Geometry* (Springer Berlin Heidelberg, 2006). URL http://dx.doi.org/10.1007/3-540-33099-2.

[17] Bakshi, A., Liu, A., Moitra, A. & Tang, E. Learning quantum hamiltonians at any temperature in polynomial time. *arXiv preprint arXiv:2310.02243* (2023).

[18] Bluhm, A., Capel, Á. & Pérez-Hernández, A. Exponential decay of mutual information for gibbs states of local hamiltonians. *Quantum* **6**, 650 (2022).

[19] Araki, H. Gibbs states of a one dimensional quantum lattice. *Communications in Mathematical Physics* **14**, 120–157 (1969). URL https://doi.org/10.1007/bf01645134.

[20] Pérez-García, D. & Pérez-Hernández, A. Locality estimes for complex time evolution in 1d. *Communications in Mathematical Physics* **399**, 929–970 (2023). 2004.10516.

[21] Kliesch, M., Gogolin, C., Kastoryano, M. J., Riera, A. & Eisert, J. Locality of temperature. *Physical Review X* **4** (2014). URL https://doi.org/10.1103%2Fphysrevx.4.031019.

[22] Pfeuty, P. The one-dimensional Ising model with a transverse field. *Annals of Physics* **57**, 79–90 (1970).

[23] Sachdev, S. *Quantum Phase Transitions* (Cambridge University Press, 2011), 2nd edn.

[24] ApS, M. *The MOSEK optimization toolbox for MATLAB manual. Version 10.0.* (2022). URL http://docs.mosek.com/10.0/toolbox/index.html.

[25] O'donoghue, B., Chu, E., Parikh, N. & Boyd, S. Conic optimization via operator splitting and homogeneous self-dual embedding. *Journal of Optimization Theory and Applications* **169**, 1042–1068 (2016).

[26] Farenick, D. R. & Zhou, F. Jensen's inequality relative to matrix-valued measures. *Journal of mathematical analysis and applications* **327**, 919–929 (2007).
